# Arc Dynamics and Erosion Behavior of Pantograph-Catenary Contacts Under Controlled Humidity Levels

**DOI:** 10.3390/s25165208

**Published:** 2025-08-21

**Authors:** Bingquan Li, Yijian Zhao, Ran Ji, Huajun Dong, Ningning Wei

**Affiliations:** 1 School of Mechanical Engineering, Dalian Jiaotong University, Dalian 116028, China; libingquan@lntdxy.edu.cn (B.L.); zhaoyijian321@163.com (Y.Z.); weiningning@dzu.edu.cn (N.W.); 2 Department of Railway Rolling Stock, Liaoning Railway Vocational and Technical College, Jinzhou 121000, China

**Keywords:** image processing, arc dynamics characteristics, relative humidity, arc erosion, current-carrying

## Abstract

In response to the instability fluctuations and erosion characteristic changes in pantograph-catenary system (PCS) arcs induced by humidity variations in an open environment, a single-variable controlled experimental approach based on multi-source data fusion is proposed. This study innovatively establishes a humidity-controlled reciprocating current-carrying arc initiation test platform, integrating digital image processing with the dynamic analysis of multi-physics sensor signals (current, voltage, temperature). The study quantitatively evaluates the arc motion characteristics and the erosion effects on the frictional contact pair under different relative humidity levels (30%, 50%, 70%, and 90%) with a DC power supply (120 V/25 A). The experimental data and analysis reveal that increasing humidity results in higher contact resistance and accumulated arc energy, with arc stability first improving and then decreasing. At low humidity, arc behavior is more intense, and the erosion rate is faster. As humidity increases, the electrode wear transitions from adhesive wear to electrochemical wear, accompanied by copper transfer. The results suggest that the arc stability is optimal at moderate humidity (50% RH), with a peak current-carrying efficiency of 66% and a minimum loss rate of 14.5%. This threshold offers a vital theoretical framework for the optimization and risk assessments of PCS design.

## 1. Introduction

The Pantograph-Catenary System (PCS) is responsible for providing a stable current collection for electrified trains. However, during train operation, the inevitable vibrations and shocks cause offline arcing phenomena, which exhibit complex and variable characteristics within the open, multi-dimensional physical field, significantly affecting the stability of train operations [1]. Unlike the flexible catenary systems used in high-speed railways, urban electrified railways employ rigid catenary systems and a direct current (DC) power supply, resulting in more pronounced corrugation wear and high-frequency vibration-induced abrasion [2]. In urban environments, due to the combined effects of infrastructure, meteorological conditions, and geographical factors, the spatial and temporal variations in factors such as humidity, airflow, temperature, and atmospheric pressure significantly influence the arc initiation threshold, energy dissipation process, and erosion characteristics of the contact surface [3]. Therefore, gaining an in-depth understanding of how environmental factors affect the dynamic behavior and erosion of pantograph-catenary arcs is crucial for developing more robust PCS designs and predictive maintenance strategies.

In recent years, extensive research has been conducted in related fields. For example, Xu et al. [4] established a dynamic model of pantograph-catenary arcs based on high-speed airflow fields, with simulations revealing a nearly linear relationship between arc dissipated power and airflow velocity. Numerous studies have investigated the effects of temperature through a series of current-carrying friction experiments. Studies [5,6] found that under low-temperature icing conditions, pantograph-catenary arcs exacerbate both electrical and mechanical wear, negatively impacting current collection quality. In contrast, studies [7,8] revealed that high-temperature interfaces significantly inhibit arc ablation and delamination wear, but intensify adhesive wear. Studies [9,10,11] combined experimental and simulation approaches to comprehensively investigate the characteristics of arcs in low-pressure environments, concluding that arcs exhibit continuous reciprocating motion under normal atmospheric pressure. However, when the pressure drops to between 25,327 and 30,689 Pa, the arcs cease movement, a phenomenon referred to as the “relative static pressure for arc motion.” As the pressure continues to decrease, the arcs undergo stable reverse motion. Currently, many scholars continue to focus on the friction and wear behavior of pantograph-catenary systems under humidity conditions. Studies [12,13,14] employing X-ray photoelectron spectroscopy (XPS) have explored the impact of humidity variations on the friction characteristics of current-carrying rolling contacts, finding that as humidity increases, the friction coefficient significantly rises, and the wear mechanism of the materials shifts from adhesive wear to layer fatigue wear. Recent studies over the past two years have further elucidated humidity-induced accelerated oxidation erosion on metallic materials. Investigations [15,16] employing multiple material characterization techniques revealed a water molecule-triggered spontaneous oxidative dispersion mechanism in metal oxide nanoparticles. Zhao et al. [17] implemented bilayer graphene doping technology to achieve oxidation resistance for copper electrodes in extreme service environments, though fabrication costs warrant further optimization. Collectively, these findings underscore the complex coupling effects between environmental factors and material electrochemical behavior. Nevertheless, a systematic understanding of arc dynamics and erosion thresholds regulated solely by spatial humidity variations remains limited.

This study investigates air humidity effects on arc motion and erosion in rigid pantograph-catenary contact pairs. An innovatively designed humidity-controlled reciprocating current-carrying test platform integrates synchronized high-speed imaging and multi-physics sensing, enabling the comprehensive capture of offline arc discharge during rigid sliding electrical friction. Digital image processing quantitatively resolved arc motion morphology while elucidating spatiotemporal evolution patterns of arc characteristics, localized temperature fields, volt-ampere properties, and current-carrying parameters under varying humidity levels. High-resolution macro-lens imaging characterized the arc-induced erosion morphology of contact interfaces and identified critical environmental thresholds affecting system longevity. Most significantly, we revealed a dual-stage mechanism governing humidity-regulated arc stability (enhancement–attenuation transition), with 50% RH significantly optimizing the current-carrying efficiency and current collection quality. These findings transcend the limitations of multiparameter-coupled studies, establishing theoretical foundations for arc dynamics risk assessment and section-specific humidity-adaptive designs (notably humidity-sensitive zones) in open-environment PCS. The results aim to enhance the resilience and reliability of urban rail transit power supply systems under variable climatic conditions.

## 2. Experimental Methodology

### 2.1. Experimental Materials

The friction pair electrode materials selected for this experiment include a standard pure copper (Cu-CATH-2) contact wire with a cross-sectional area (S) of approximately 120 mm^2^ and a pure carbon plate with a carbon content of no less than 98%. To meet the requirements for contact wire profile experiments, the tip of the pure copper contact wire has been processed to achieve a curvature radius (ρ) of approximately 6 mm.

The curvature radius is defined as Equation (1):

(1) $$\rho =\frac{1}{K}=\frac{{\left(1+{\dot{y}}^{2}\right)}^{\frac{3}{2}}}{\left|\ddot{y}\right|}$$

where *K* is the curvature, and *y* is the curve equation with the second-order derivative.

**Figure 1:**
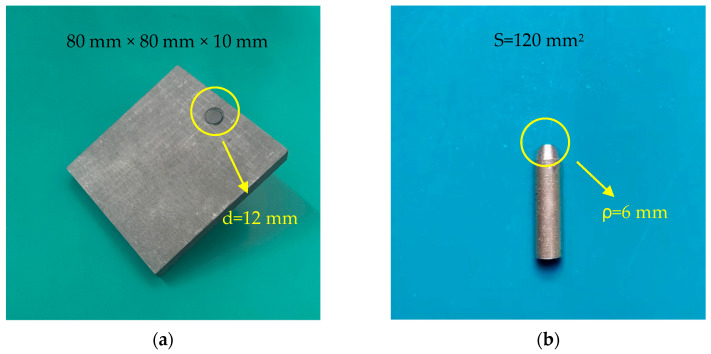
The friction pair electrode samples. (**a**) Pure carbon plate. (**b**) Pure copper rod (Cu-CATH-2).

To facilitate the observation of the arc discharge process, the carbon sliding plate was fabricated into a square shape with dimensions of 80 mm × 80 mm × 10 mm. A threaded hole with a diameter (d) of 12 mm was made at the top, and a 25 mm-long carbon rod of the same material was embedded. Figure 1 shows the friction pair electrode samples selected for this experiment, which are fixed in opposing horizontal positions using insulated epoxy resin plates, mounted on two vertically arranged dual-axis linear slides.

The detailed physical properties of the experimental electrodes are shown in Table 1.

### 2.2. Experimental Platform

The experimental platform developed in this study features a carefully designed electrical circuit, control circuit, and data acquisition (DAQ) system to thoroughly investigate the complex characteristics of arc dynamics in the rigid pantograph-catenary arc discharge scenario. The entire experimental setup primarily includes an adjustable DC power supply, adjustable resistors, an arc generation chamber, an arc monitoring and control system, a high-speed camera, and an electrode humidifier. The adjustable DC power supply (IT6018D-1500-40, ITECH, China) in this platform simulated the test voltage, delivering a maximum output of 1500 V DC with ≤0.02% full-scale error; concurrently, the adjustable load resistor regulated the current magnitude within the aforementioned electrical circuit to ensure operational safety and reliability.

To simulate the “zig-zag” sliding motion between the pantograph and catenary, the arc generation chamber was modified from conventional arc fault generators (AFGs), a key innovation of this study. The upgraded chamber primarily utilized a stepper motor as the driving source, with precision control of two orthogonally stacked dual-optical-axis linear slides (Slide 1 is positioned at the bottom, and Slide 2 is positioned at the top, which are abbreviated as S1 and S2, respectively) via a digital driver, step controller, and limit switches. A copper rod (fixed electrode) was mounted on Slide 1, while a carbon plate (movable electrode) was affixed to Slide 2’s carriage block. Through programmed instructions to the digital driver, the stepper motor was controlled to achieve both offline and sliding two-dimensional displacement of the movable electrode at preset velocities. To facilitate better observation of the arc ablation process with the high-speed camera, an opaque PET insulation film (with a light transmittance of λ ≤ 5%) was applied around the arc generation chamber to minimize interference from external factors.

The arc monitoring and control system was designed to adjust various experimental parameters, including electrode contact pressure, inter-electrode offline velocity, loop current, and humidity levels. Additionally, it could collect and store signals such as inter-electrode voltage, current, temperature, and environmental humidity. The measurement instrumentation comprised the following:

- Hall current sensor (CHB50-SF, SENSOR Electronics, China)
- Hall voltage sensor (CHV-50P, SENSOR Electronics, China)
- K-type thermocouples
- Temperature-humidity sensor
- Thin-film pressure sensor

Current sensors were installed on the movable electrode side of the electrical circuit to measure inter-electrode current variations, with a maximum range of 50 A and ≤0.8% error. Voltage sensors were wired to the roots of the copper rod and carbon plate, respectively, monitoring inter-electrode voltage fluctuations up to 1000 V (≤0.8% error). K-type thermocouples measured temperatures at multiple locations on the carbon plate (max. 1000 °C, ≤0.4% error). The temperature-humidity sensor was wall-mounted inside the arc generation chamber to monitor environmental stability (≤2% RH error). A thin-film pressure sensor cooperated with the step controller to ensure a reliable initial electrode connection.

**Figure 2:**
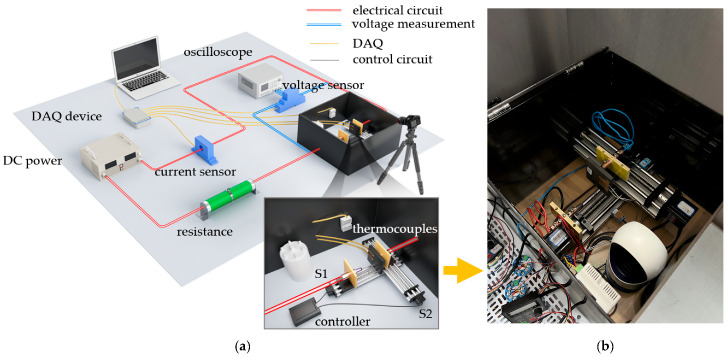
The schematic of the experiment apparatus. (**a**) Electrical circuit, control circuit and DAQ. (**b**) Arc generation chamber.

All measurement data underwent online high-frequency sampling through a DAQ device (USB-3123, Smacq, China) with a maximum sampling rate of 1 MSa/s. Figure 2 shows the schematic of the experimental apparatus used in this study.

In this experimental apparatus, an adjustable electrode humidifier was installed within the enclosed arc generation chamber to create the required environmental humidity conditions, with temperature and humidity values continuously monitored by sensors and fed back to the control system. Additionally, two temperature monitoring points (thermocouples) were set on the pure carbon plate electrode material, fixed at 20 mm and 40 mm above the contact interface, labeled as TH and TL, respectively. These points are used to capture the temperature field changes in the vicinity of the arc during its evolution. A polarizing filter was installed on the high-speed camera lens to block interfering light, with a frame rate of 1250 fps and a resolution of 720 pixels (px) × 720 pixels (px) to capture the pantograph-catenary arc discharge process. An auxiliary voltage signal channel was created, allowing the DAQ to synchronize and transmit signals to the camera shutter as the sensor data collection begins, ensuring correspondence between the video timestamp and the data acquisition timestamp. Notably, during the entire experiment, the DAQ system continuously collects experimental data, including arc voltage, arc current, and temperature field, at a sampling frequency of 1 kSa/s.

### 2.3. Experimental Conditions

The experimental conditions for the pantograph-catenary arc characteristics are shown in Table 2. The ambient temperature was maintained at 25 °C. According to the National Statistical Yearbook of China, the average relative humidity (RH) in major cities ranges from 30% RH to 90% RH. Therefore, the experimental humidity levels were controlled at 30% RH, 50% RH, 70% RH, and 90% RH. To ensure experimental repeatability and ablation feature uniformity, preliminary tests established optimal motion parameters: 10 mm/s sliding velocity and 5 mm/s offline velocity. As Ref. [18] identifies the “zig-zag” sliding motion as a critical arc-triggering factor, all experiments employed a fixed unidirectional motion path. Limit switches ensured consistent starting positions to minimize mechanical errors affecting ablation morphology.

Before the experiment, the surfaces of the friction pair electrode materials (pure copper rod and pure carbon plate) were polished to ensure smoothness. The copper rod was connected as the anode to the positive terminal of the DC power supply, and the carbon plate was connected as the cathode to the negative terminal. Both electrodes were fixed in opposing directions on two orthogonal dual-axis linear slides, with the sides of the two electrodes aligned within the same vertical plane to facilitate accurate focusing by the high-speed camera. Using a step controller, the initial contact pressure between the electrodes was precisely applied at 50 N, and a multimeter was used to measure the contact resistance (*R*), which was maintained at *R* ≤ 10 mΩ to ensure a good electrical contact. To confirm the accuracy of subsequent data processing, the resistance of the frictional contact electrodes was measured at 25 °C prior to the experiment and verified during the testing process.

Yao et al. [19] investigated DC arc characteristics through repeated experiments under 75 V to 300 V and 6 A to 30 A conditions, revealing that voltage exerted negligible influence on arc resistance whereas load current demonstrated significant effects. Comparative analysis confirmed stable arc combustion and consistent volt-ampere properties at 25 A. Following humidity stabilization within the arc generation chamber, the variable resistor was adjusted to achieve target loading, enabling a DC power supply output at 120 V/25 A. After activating the power supply and stabilizing the electrical circuit, the movable electrode’s velocity parameters were set to simulate pantograph-catenary sliding separation, thereby triggering arcing. Once the arc extinguished, the power supply was quickly turned off, and the electrode material ablation was recorded. The experimental samples were preserved, and the sensor data was archived.

In accordance with the designed humidity level variables, the tests were repeated three times under consistent experimental conditions, with a 30-minute interval between each test to allow the electrodes to cool down. Additionally, the electrode materials were polished to remove any residual oxidation layer or ablation marks from previous experiments, minimizing the influence of random experimental variations.

## 3. Results and Discussion

### 3.1. Arc Morphology and Its Evolution

**Figure 3:**
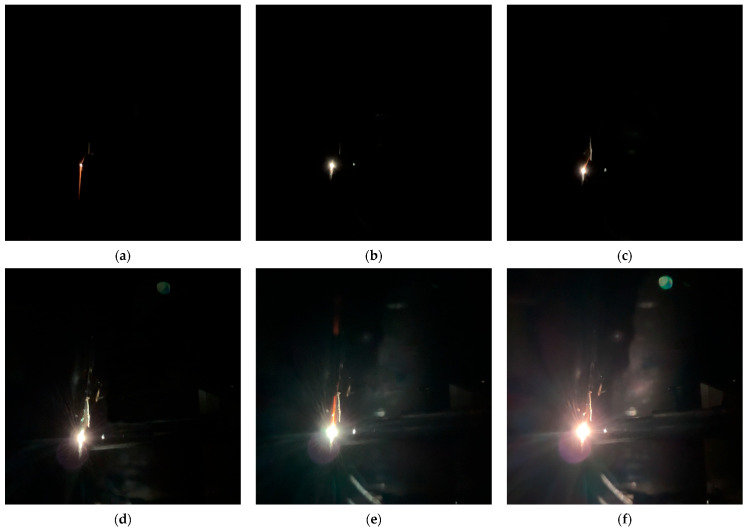

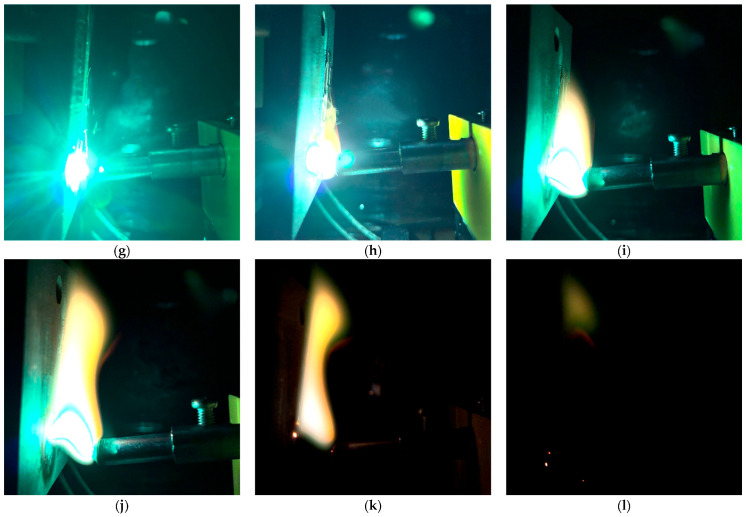
Arc evolution process: (**a**) t = 120 ms; (**b**) t = 160 ms; (**c**) t = 200 ms; (**d**) t = 240 ms; (**e**) t = 320 ms; (**f**) t = 400 ms; (**g**) t = 560 ms; (**h**) t = 1280 ms; (**i**) t = 2000 ms; (**j**) t = 2320 ms; (**k**) t = 2360 ms; (**l**) t = 2400 ms.

In Table 2, repetitive experiments were conducted under different humidity levels. Experimental observations showed that under various humidity levels, the arc morphology evolution tended to follow a consistent process. Figure 3 illustrates the complete arc morphology evolution process under 70% RH, which can be divided into four main stages:

- Arc Triggering Stage: During the relative sliding contact motion of the friction pair electrode materials, friction causes the carbon plate contact surface to become microscopically uneven. As the pantograph-catenary system moves offline, the contact pressure between the electrodes decreases, and the electric field intensity between the electrodes meets the gas breakdown conditions, triggering the arc. As shown in Figure 3a–c, the arc exhibits a longitudinal stretching trend during the sliding contact.
- Arc Diffusion Stage: Under the combined effects of the electric field force and thermal diffusion, the arc rapidly expands outward, transforming from an irregular light spot into a single elliptical shape. As shown in Figure 3d–f, the arc area and brightness increase continuously, accompanied by significant changes in the current and voltage between the electrodes.
- Arc Stabilization Stage: As the input current stabilizes, the arc enters a steady combustion phase. At this stage, a large amount of heat is generated between the friction pair, and factors such as contact wire vibration and material irregularities cause the deformation of the arc column. The arc root undergoes displacement, and the arc light appears blue-green, indicating the formation of a large number of carbon-containing free radicals during combustion. As shown in Figure 3g–i, the single elliptical arc undergoes elongation and twisting, with the arc root moving in the longitudinal direction.
- Arc Extinction Stage: As shown in Figure 3j–l, the arc continues to stretch, and the arc light area increases as the plasma density decreases. As the current weakens instantaneously, the arc energy sharply decreases, causing the arc column to shrink and the arc light to dim, leading the arc to gradually extinguish. This stage occurs relatively quickly.

### 3.2. Arc Intensity and Temperature Characteristics

**Figure 4:**
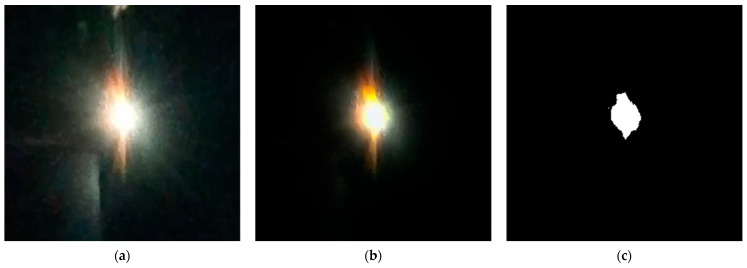
Arc image processing. (**a**) 260 px × 260 px image. (**b**) Image denoising. (**c**) Binary image.

To quantitatively evaluate the arc intensity characteristics, the batch preprocessing of arc images captured by high-speed imaging systems is essential. As illustrated in Figure 3, the arc images are affected by various environmental factors, such as ionized material motion, leading to the presence of significant noise and blurring of the arc’s boundary. Figure 4a presents the image cropped to a 260 px × 260 px resolution. In Figure 4b, the image is processed using wavelet transformation for noise reduction, in conjunction with the Curvelet transform algorithm, which substantially enhances the definition of the arc’s edge. Following binarization preprocessing, as shown in Figure 4c, the high-quality image enables dynamic quantification of arc intensity through statistical summation of pixel values within arc contours (equivalent to arc area). This methodology has been applied and validated in prior studies by our research group [20]. Analysis of gray-level distributions in Figure 4c reveals that pixel values of 255 (arc region) predominate, while gray-level 0 corresponds to the background. Other values exhibit irregular distributions with predominantly zero counts, attributable to image noise and thus excluded from detailed analysis. Consequently, this study adopts the 255 gray-level threshold as the definitive arc intensity metric for extracting dynamic morphological features.

**Figure 5:**
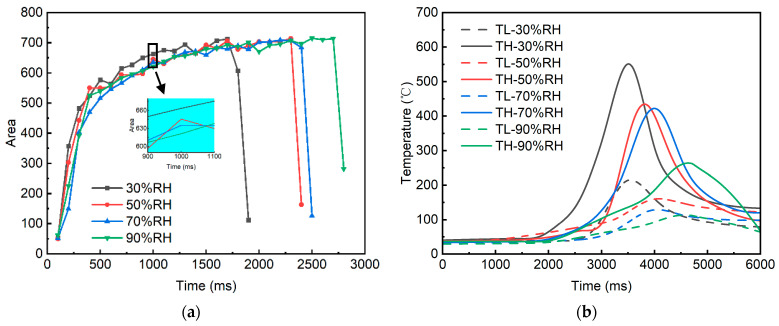
Variation in arc area and temperature in the vicinity of the arc with humidity levels. (**a**) Arc area. (**b**) The temperature in the vicinity of the arc.

The arc intensity varies throughout the arc evolution process and is influenced by the thermal effects at the pantograph-catenary contact interface, including frictional heat and Joule heating [21,22]. To accurately characterize the arc intensity, this study records the temperature variations on the carbon plate material surface under different humidity conditions and extracts image sets from the complete arc morphology evolution for preprocessing. The high-speed imaging system software exports the image sets at a predefined frame rate, with a frame rate of 10 fps selected to enhance computational speed, and calculations are performed using Matlab 2024b. Figure 5 depicts variations in arc area and arc vicinity temperature fields under identical experimental conditions across different humidity levels.

Figure 5a illustrates that under varying humidity conditions, the arc area consistently increases throughout the evolution process, with the rate of expansion transitioning from rapid to gradual. A detailed inspection at a specific moment reveals that, at lower humidity levels, the arc exhibits a slightly larger morphology. As analyzed in Figure 5b, this phenomenon can be primarily attributed to the relatively lower thermal conductivity of dry air at reduced humidity. This leads to the maintenance of higher temperatures in the vicinity of the arc, which in turn results in an enhanced thermal expansion of the plasma in the arc region. Consequently, the morphology of the arc root and tail expands, forming a comparatively larger arc configuration. Conversely, as the environmental humidity increases, the higher thermal conductivity provides a more efficient medium for heat dissipation, thereby concentrating on the “hot tip” of the arc. This causes the arc morphology to become narrower, leading to a reduction in the arc column area. Table 3 summarizes the experimental results of the extreme values of TH and TL under different humidity levels, along with their respective differences, which characterize the temperature variation per unit distance. Notably, although the increase in humidity enhances the thermal conductivity of the arc region, facilitating a more pronounced cooling effect, it also induces a more uniform distribution of the temperature field within the arc. This results in a relatively thermally stable state of the arc. This could explain the observed increase in arc duration, although it is important to note that the heat accumulation effects within the enclosed experimental space may have contributed to potential deviations in the results.

### 3.3. Arc Discharge Characteristic

**Figure 6:**
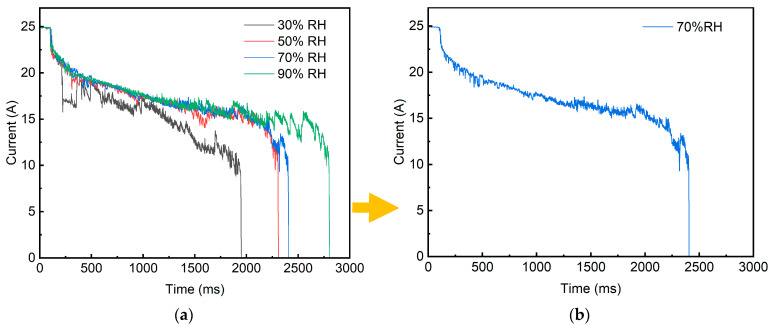
Arc current curve of pantograph-catenary under various humidity levels. (**a**) The results of the first repeated experiments. (**b**) 70% RH.

**Figure A1:**
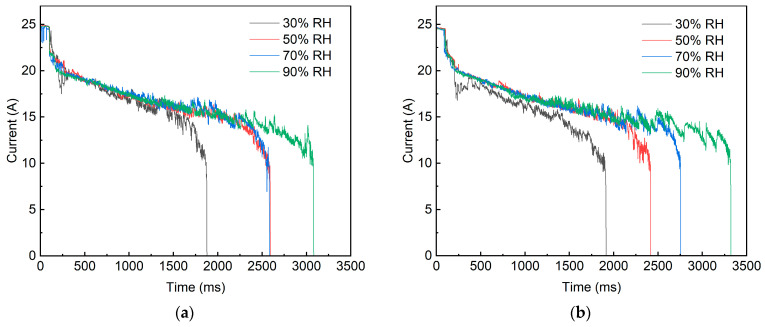
Arc current curve of pantograph-catenary under various humidity levels. (**a**) The results of the second repeated experiments. (**b**) The results of the third repeated experiments.

One of the primary methods for characterizing the dynamic properties of the arc is by observing the electrical performance during arc combustion, which directly determines the current collection quality and current-carrying stability of the pantograph-catenary system. The variations in current and voltage between the electrodes are directly measured through sensors. Since current and voltage are interdependent, their trends exhibit a mirror-symmetric relationship. Therefore, Figure 6 illustrates the changes in the pantograph-catenary current waveform under different humidity levels, as measured under the experimental parameters specified in Table 2. Figure 6a displays the experimental results of the first repetition. For clarity, the current waveform curve at a single humidity level (70% RH) extracted from this initial experiment is presented in Figure 6b. Figure A1a displays the results of the second experiment, while Figure A1b shows the results of the third experiment.

Based on the arc morphology evolution process shown in Figure 3, taking 70% RH as an example, the typical time intervals in Figure 6b can be analyzed as follows:

- 0–100 ms: The frictional electrode materials maintain good sliding contact, and the current value remains at 25 A.
- 100–200 ms: The electrode materials go offline, and the arc is triggered instantaneously, with the current rapidly decreasing to 22 A.
- 200–480 ms: During the arc diffusion phase, the current shows “intermittent spiking” with a decelerating rate of change. This phase represents the first unstable arc combustion stage, where sparks, as shown in Figure 4a, are observed.
- 480–2320 ms: The arc current fluctuates in a relatively stable pattern, with the rate of change slowing down again. This phase releases a substantial amount of heat, with the current dropping to 15 A. From 2180 ms onwards, the arc begins to diffuse again, and the current experiences significant fluctuations, with a maximum drop of 9 A, indicating the arc reignition phenomenon.
- 2320–2400 ms: The arc extinguishes abruptly, and the current sharply decreases from 13 A to 0 A.

Figure 6 demonstrates that different environmental humidity levels exert a significant influence on arc current waveforms. Specifically, at 30% RH, a significant regulatory effect on the arc diffusion stage is observed. At 200 ms, the arc current curve exhibits a “cliff-like” fluctuation, lasting for 200 ms, with the arc image showing frequent arc pulling phenomena. This may be due to the lack of a conductive water film on the electrode material surface under the influence of low humidity, leading to carbon accumulation and oxidation, forming a micro-insulating layer. This results in poor contact, causing a local increase in contact voltage and repeated arc pulling.

From Figure 6, it can be observed that 50% RH has a slight effect on the early diffusion stage of the arc, but the current fluctuations are not as severe, lasting 100 ms. Notably, during the periods from 1550 to 1700 ms and 1900 to 2200 ms, short-term current fluctuations occur in the stable combustion phase, with the arc image showing a longitudinal movement of the arc root. This may be attributed to the increased humidity, which enhances the arc’s cooling effect, causing a short-term decrease in current.

Compared with the condition at 70% RH, the arc combustion duration is longer and the fluctuation of the arc current is more stable under 90% RH. However, towards the end of the stable combustion phase, between 1800 and 2700 ms, the current exhibits large and frequent periodic fluctuations until the arc extinguishes. This phenomenon could be due to the significantly increased air conductivity in high-humidity environments, allowing the arc to form and maintain stability instantly. Furthermore, the enhanced thermal conductivity induced by humidity variations leads to a rapid decrease in the temperature near the arc, triggering periodic current attenuation. Meanwhile, gas flow disturbances generated by the evaporation of water films on the electrode surface further amplify the fluctuation amplitude. Study [16] confirms that surface hydroxyl (OH) groups play a significant role in governing the redispersion of metal nanoparticles (NPs). Consequently, under high humidity levels, metal particles on the electrode surface are efficiently converted into a dispersed ionic state. These ions, together with electrolyzed hydroxyl ions, are co-adsorbed by water molecules to form ion-hydrated molecular clusters. This process affects the longitudinal migration of plasma between the electrodes, resulting in frequent inter-electrode disturbances, repeated arc ignitions and extinctions, and ultimately manifesting as sustained undulatory fluctuations in the current.

It is important to note that all of the above experimental results are based on a closed experimental space, and the possibility of sealed space effects on the experimental outcomes cannot be excluded. Although current curve fluctuations primarily originate from the stochastic nature of arcing, systematic errors warrant consideration. Primary error sources include the following:

- DAQ quantization error: High-frequency sampling by the DAQ may exhibit minor serrated fluctuations in current waveforms due to signal transmission quantization.
- Motion precision error: Minor trajectory deviations during sliding-offline transitions on the biaxial orthogonal slides may cause instantaneous current spikes.
- Sensor intrinsic error: While individual sensor errors remain within acceptable limits (≤0.8%), cumulative error propagation could influence arc evaluation metrics. Calculated cumulative effects fall within tolerable thresholds.

Comparative analysis of current curves from triplicate experiments under identical humidity levels demonstrates strong consistency in trend patterns. This confirms that systematic errors do not significantly interfere with fundamental arc characteristics, establishing the statistical reliability of the experimental outcomes.

This study also introduces four evaluation parameters to assess the arc discharge characteristics and current-carrying quality: contact resistance, cumulative arc energy, current-carrying efficiency, and current-carrying stability. Experimental measurements at 25 °C confirmed that the total resistance of the contact pair accounted for 2.56% of the preset variable resistance value. However, dynamic resistance variations occurred due to the electrode temperature rise during testing. To simplify calculations and minimize systemic errors, this study adopts a consistent approach by excluding dynamic resistance variations from the model.

Contact resistance is defined as

(2) $$R_{c}=\frac{1}{n}\sum_{i}^{n}\frac{U_{i}}{I_{i}}-R_{e}$$

In this equation, $R_{c}$ represents the average contact resistance between the electrode contacts, $R_{e}$ is the measured total resistance of the initial friction pair electrodes, $U_{i}$ is the instantaneous arc voltage between the friction pair electrodes, and $I_{i}$ is the instantaneous arc current at the corresponding time.

Cumulative arc energy is an important reference for evaluating arc heat, and it is defined as

(3) $$E=\int UI-I^{2}R_{e}dt$$

where U is the voltage drop between the friction pair electrodes, and I is the circuit current, with the integration interval covering the entire duration of the experiment [23].

Current-carrying efficiency is a measure of the current-carrying quality, and it is defined as

(4) $$\eta =\frac{\bar{I}}{I_{0}}\times 100\%$$

where $\bar{I}$ is the average current during the experiment, and $I_{0}$ is the test current value (25 A in this study).

The current-carrying stability determines the energy loss efficiency and conversion capability. Current-carrying stability is defined as

(5) $$\delta =\frac{\sqrt{\frac{1}{n}\sum_{i=1}^{n}{\left(I_{i}-\bar{I}\right)}^{2}}}{\bar{I}}\times 100\%$$

where δ is a dimensionless parameter [24].

**Figure 7:**
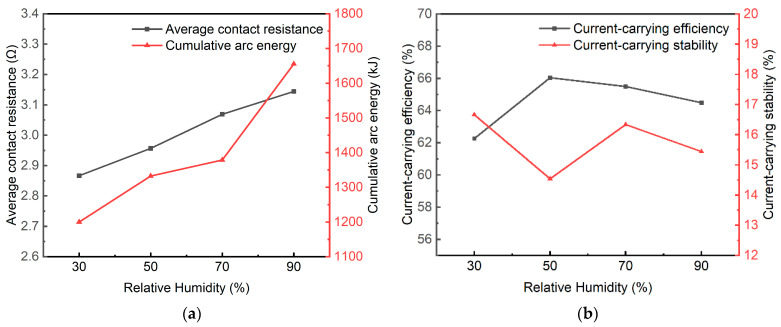
Arc evaluation quality affected by various humidity levels with the test parameters in Table 2. (**a**) Average contact resistance and cumulative arc energy. (**b**) Current-carrying efficiency and current-carrying stability.

Figure 7 shows the trend of the arc evaluation quality parameters of the pantograph-catenary system under varying environmental humidity in the coupled experiments listed in Table 2.

Figure 7a demonstrates that as the humidity increases, both the average contact resistance and accumulated arc energy show an increasing trend, reaching their maximum values at 90% RH. Study [25] revealed that elevated humidity transforms condensation behavior on hydrophilic (copper-based) surfaces from dropwise to film-wise mode. Consequently, the underlying mechanism for this phenomenon may be attributed to the increase in water vapor content in the air, which leads to the formation of a condensed water film on the electrode surface. After partial electrolysis, this water film, in conjunction with the surface oxide film formed by temperature elevation, collectively forms a composite surface film. Although this surface film provides lubricating and friction-reducing effects, it also increases contact resistance, negatively impacting the current-carrying stability. Furthermore, from Equation (3), it is evident that accumulated arc energy is positively correlated with contact resistance, indicating that in a humid environment, the frequency of arcs generated between the electrode materials is higher, resulting in more accumulated arc energy.

Analyzing Figure 7b, it can be observed that the current-carrying efficiency (η) at 50% RH is the highest at 66%, while the current-carrying stability (δ) is as low as 14.5%, indicating the optimal current-carrying quality and lowest energy loss. In contrast, at 30% RH, η is the lowest at 62%, indicating a decrease in energy carrying efficiency, and δ rises to 17%, suggesting instability in the current. Interestingly, from the 50% RH onwards, as the humidity increases, η tends to decrease while δ increases, indicating a decline in current-carrying quality. However, at 90% RH, δ shows a significant decrease, indicating a reduction in instability. This suggests the presence of an “optimal mid-humidity range” for pantograph-catenary arcs, within which arc stability is enhanced, arc frequency is lower, energy loss is minimized, and current transmission stability in the pantograph-catenary system is superior. The experimental results indicate that the range around 50% RH is the “optimal humidity range”.

### 3.4. Erosion Characteristics of Carbon Electrodes

**Figure 8:**
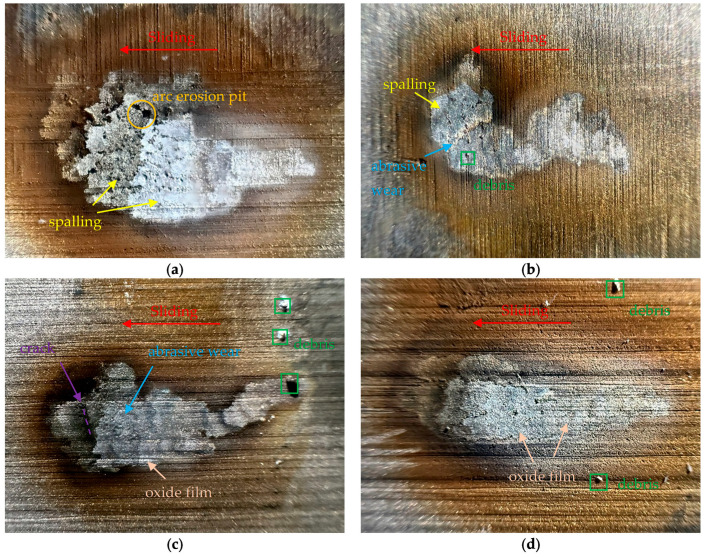
Image of the arc ablated traces: (**a**) 30% RH; (**b**) 50% RH; (**c**) 70% RH; (**d**) 90% RH.

Figure 8 shows the surface morphology of the friction pair electrodes (pure carbon plates) after erosion under different humidity levels. In general, it is observed that the electrode material experiences arc erosion wear primarily at the trailing edge of the sliding offline process. Along the direction of motion, the surface quality gradually deteriorates. Furthermore, arc erosion exhibits a characteristic of dynamic longitudinal migration.

As observed in Figure 8a, under 30% RH, the arc sliding erosion marks are wider, and large-scale delamination and erosion pits are observed in the molten pools. This phenomenon is primarily caused by the combined effect of frictional heat between the electrode materials and the Joule heat generated by the arc, leading to adhesive wear and subsequent crack formation and delamination on the carbon material contact surface [26]. An additional potential mechanism is that the absence of a lubricating film on the electrode surface under low humidity levels, coupled with microscale surface roughness, results in poor contact between the contact pairs [27]. This induces a “current agglomeration effect,” where heat accumulates locally, leading to uneven mechanical stress distribution on the electrode surface. Figure 8b reveals a small amount of abrasive wear, with the arc erosion marks becoming narrower and longer as humidity increases. In all four humidity experimental environments, small metallic debris was found on the material surface. As shown in Figure 8c, this could be due to copper electrode droplets formed under high-temperature conditions in the arc [28], which splashed onto the electrode material sliding path. It was also found that, with increasing humidity, erosion pits on the material surface decreased, and the wear type shifted primarily to minor abrasive wear and fine scratches. This could be attributed to the formation of a lubricating film on the electrode material surface due to increased ambient moisture [29].

In comparison, under high humidity experimental conditions, the carbon material surface exhibits white flaky particles accumulated as an oxide layer, as shown in Figure 8d. This could be a metal oxide film formed by the frictional sliding of copper electrodes under the experimental conditions. The increase in humidity promotes the electrochemical oxidation of the metal, indicating the occurrence of copper transfer in this experimental condition. The corresponding electrochemical reaction can be represented as [30]

Cu + H_2_O → CuO + 2H^+^ + 2e^−^. (6)

Due to the reduced conductivity of copper oxide compared to copper electrodes [31], this further explains the increase in arc contact resistance with the rise in humidity.

In brief, based on the experimental data, this study demonstrates that variations in humidity predominantly have complex effects on the physical and discharge characteristics of the arc, with these effects largely dependent on the cooling effects of frictional heat and Joule heat from the electrode materials. Additionally, high humidity environments promote the formation of lubricating and electrochemical oxide films on the electrode surfaces, significantly increasing contact resistance and accumulated arc energy, which in turn has a profound microstructural effect on material erosion. The findings highlight the environmental sensitivity of pantograph-catenary system offline arcs, providing valuable insights for risk prediction related to pantograph system failures or the coupling effects of complex environmental factors.

## 4. Conclusions

This study experimentally investigates the arc motion discharge and erosion characteristics of PCS contact pair electrodes under different humidity levels. The effects of humidity on the arc’s physical properties (arc intensity and near-arc temperature) and discharge characteristics (voltage-current characteristics, contact resistance, accumulated energy, current-carrying efficiency, and stability) are discussed. The erosion damage characteristics of electrode materials under varying humidity are compared and analyzed. Through comparative validation with prior studies, the core findings are summarized as follows:

1. Arc Morphology Evolution: The arc morphology evolution exhibits a tendency toward convergence. As the humidity increases, the arc’s “thermal tip” becomes more concentrated, and the arc column shape narrows, resulting in a more uniform temperature distribution in the arc vicinity and enhanced arc persistence.
2. Arc Current Behavior: The arc current shows an overall “intermittent” decreasing fluctuation trend. As humidity increases from 30% RH to 90% RH, the region of intense current fluctuation gradually shifts from the early to the late stages of the arc development. At medium humidity levels (50% RH, 70% RH), current fluctuations are the most stable, indicating the most stable arc combustion.
3. Impact on Electrical Performance: High humidity significantly increases both contact resistance and accumulated arc energy. This conclusion is consistent with the experimental results of Song et al., which found that increasing surface humidity reduces the conductivity of electrode materials [14]. However, in terms of current-carrying quality, the current-carrying efficiency reaches its peak at 50% RH (66%), while the current-carrying stability (loss rate) is lowest at 14.5%, indicating the optimal current-carrying quality. This suggests the presence of an “optimal arc combustion zone” at 50% RH.
4. Material Erosion Analysis: Based on material erosion observations, the arc root exhibits a longitudinal motion migration characteristic. As humidity increases, the arc erosion marks become narrower and longer. At low humidity, electrode material erosion is primarily characterized by abrasive and adhesive wear. At high humidity, the participation of water vapor leads to a transition towards electrochemical oxidation wear, accompanied by copper material transfer phenomena. This specific conclusion exhibits partial similarity to the findings of Fan et al. [16], where water molecules and oxygen jointly induce the formation of hydroxylated copper species from copper nanoparticles, thereby accelerating the migration of copper atoms across metal support surfaces.

In contrast to emerging graphene coatings offering long-term protection in extreme environments [17], the 50% RH threshold identified in this study offers a more cost-effective environmental regulation approach for uncoated rigid PCS. Future efforts will expand the observational range of humidity levels to precisely quantify the boundary conditions of the optimal humidity window, while simultaneously investigating the profound implications of this humidity threshold on pantograph-catenary arcing under alternating current (AC) power supply scenarios.

## Figures and Tables

**Table 1:** Physical properties of the experimental electrode *.

Material Parameters	Pure Carbon	Cu-CATH-2
Hardness (10^7^ N·m^−2^)	67	96
Specific Heat Capacity (J·kg^−1^·K^−1^)	710	380
Density (kg·m^−3^)	1.82	8.9
Electrical Resistivity (µΩ·m)	12	0.017
Thermal conductivity (W·m^−1^·K^−1^)	90	390
Bending Strength (MPa)	48.1	/

* The operating temperature of the material is 25 °C.

**Table 2:** Experimental conditions.

Temperature (°C)	Humidity (% RH)	Offline Velocity (mm/s)	Sliding Velocity (mm/s)	Initial Contact Pressure (N)
25	30, 50, 70, 90	5	10	50

**Table 3:** The experimental results of TH_max_ and TL_max_.

Humidity (% RH)	TH_max_ (°C)	TL_max_ (°C)	TH_max_-TL_max_ (°C)
30	551	215	336
50	434	160	274
70	394	129	265
90	288	120	168

## Data Availability

The original contributions presented in the study are included in the article; further inquiries can be directed to the corresponding author.

## Abbreviations and Nomenclature

The following abbreviations and nomenclature are used in this manuscript:

PCS	Pantograph-Catenary System
DC	Direct Current
AC	Alternating Current
RH	Relative Humidity, %
XPS	X-ray Photoelectron Spectroscopy
Cu-CATH-2	Cathode copper
AFGs	Arc Fault Generators
PET	Polyethylene Terephthalate, the chemical formula is (C_10_H_8_O_4_)_n_
DAQ	Data acquisition
TH	Temperature at a height of 20 mm on the carbon-copper electrode contact surface, °C
TL	Temperature at a height of 40 mm on the carbon-copper electrode contact surface, °C
px	Pixels
NPs	Nanoparticles
